# Dose rate correction of a diode array for universal wedge field dosimetric verification

**DOI:** 10.1002/acm2.70050

**Published:** 2025-02-19

**Authors:** Linyi Shen, Mengyang Li, Guiyuan Li, Xinyuan Chen, Shouping Xu, Jianrong Dai, Yuan Tian

**Affiliations:** ^1^ National Cancer Center/National Clinical Research Center for Cancer/Cancer Hospital Chinese Academy of Medical Sciences and Peking Union Medical College Beijing China

**Keywords:** dose rate correction, dose verification, MapCHECK 3, universal wedge field

## Abstract

**Purpose:**

To study the performance of MapCHECK 3 (MC3) in measuring universal wedge fields and propose a dose rate correction strategy to improve MC3 measurement accuracy.

**Materials and methods:**

Universal wedge fields with different wedge angles and field sizes were measured at different depths using MC3. Considering the more prominent dose rate dependence of type 4 diodes equipped by MC3, a program was developed to automatically correct the measurements based on the instantaneous dose rate (IDR) correction curve. Central axis (CAX) doses and off‐axis doses along the wedge direction, with and without the correction, were compared with those measured by an ion chamber under the same condition. Measurements using MC3 with and without correction were also compared with the planned doses calculated by the treatment planning system (TPS).

**Results:**

If MC3 was used for universal wedge field measurement with the dose calibration factor (DCF) derived from a reference open field, an error of up to −2.4% would be introduced into the CAX dose. Other factors (field size and measurement depth) would also affect the accuracy of measurement when they differed from the absolute dose calibration and the maximum error was up to −2.9%. While greater errors were observed in the off‐axis doses at the heel side of the wedge compared to the toe side due to the greater effective thickness of the wedge inserted into the beam. After dose rate correction, the deviations in the CAX dose were reduced to within ± 1.5%. The average gamma pass rate was also improved to over 99.5%.

**Conclusion:**

Because of the more prominent dose rate dependence of type 4 diodes, MC3 is not suitable for universal wedge field measurement using the methodology for open field measurement. The correction strategy proposed in this study is convenient and can improve the accuracy of universal wedge field measurement using MC3.

## 1 INTRODUCTION

The Elekta LINAC is commonly equipped with a universal wedge filter, a beam‐modifying device that gradually reduces radiation intensity across the beam. It has been widely used in clinical radiotherapy for different sites of tumors, such as breast tumors, brain tumors, head and neck cancer, and so on.^1^, ^2^, ^3^, ^4^, ^5^, ^6^ Any effective wedge angle in the range of 0°–60° can be achieved by combining the motorized physical wedge of 60° field and the open field with a certain proportion. Like the open field, the delivery accuracy of the universal wedge field should also be verified by comparing the measured dose and planned dose calculated by the treatment planning system (TPS). So the measurement must be accurate. Otherwise, it can mislead medical physicists into making incorrect judgments regarding the status of the accelerator and the direction of model adjustment.

Some studies have employed ion chambers for the beam model commissioning of wedge fields. While this method is considered to be most accurate, it is also time‐consuming as it requires dose measurement point‐by‐point dose measurement.^7^, ^8^ Conversely, alternative studies have suggested that film should be utilized to measure the dose distribution of wedge fields. However, its accuracy and reproducibility are closely tied to the calibration and processing conditions of the film.^9^, ^10^, ^11^ In recent years, two‐dimensional (2D) diode detector arrays such as MapCHECK and MapCHECK 2 have become widely used in dosimetric verification due to their convenience and high spatial resolution.^12^, ^13^, ^14^, ^15^, ^16^

Before using any model of MapCHECK to measure different sizes of fields at different output dose rates and different depths, the dose calibration factor (DCF) and array calibration factor (ACF) should be established following the manufacturer's instructions, with a field of a certain size (e.g., 10 × 10 cm^2^) and a certain output dose rate (e.g., the maximum output dose rate of the LINAC) at a certain depth (e.g., 10 cm below the phantom surface), respectively. ACF was used to correct the sensitivity variation among diodes. DCF was determined by comparing the counts collected by the central detector in the absolute dose calibration under a certain condition with the user‐input known dose and used for converting counts collected by the diodes during the measurement to the dose.

However, recent studies have found that the type 4 diodes assembled by MapCHECK 3 (MC3) have a prominent dose rate dependency compared with type 1 and type 2 diodes equipped in MapCHECK and MapCHECK 2.^17^, ^18^ The variation of the measured dose rate of the diodes resulted from various factor (such as field size, measurement depth, etc) between calibration and measurement will introduce non‐negligible error to the measurement results. Compared with other factors, the wedge inserted into the beam would induce more significant differences in measured dose rate between calibration and measurements, not only for central diode but also for the diodes along the wedge direction. It is recommended to use open field for absolute dose calibration in the user manual. Because of the dose rate dependence of the diodes, there may be significant deviations in the measurement dose of a universal wedge field with arbitrarily equivalent wedge if the only absolute dose calibration file for the Open Field (DCF_open_) was used to correct the collected counts from both open field and wedge field. The DCF derived from the reference wedge field (e.g., a 10 × 10 cm^2^ wedge field) with the same wedge angle as the measurement wedge field can reduce the impact of wedge insertion on measurement results. However, the equivalent wedge angle used in the clinical settings varied based on clinical needs. This means that, to acquire accurate measurements, dose calibrations have to be carried out for each wedge field with a specific angle. This process is time consuming and not conducive to clinical application. Additionally, considering that for the universal wedge, the wedge field of an arbitrary wedge angle within the range of 0°–60° is constituted by combining the motorized physical wedge of 60° field and the open field with a certain proportion. It is inappropriate to adopt the correction methods proposed by previous study^18^ for wedge field measurement because the counts collected by the diodes for the wedge field component and the open field component evidently ought to be rectified using different correction factors.

The user manual of MC3 provided by SunNuclear does not explicitly state how to perform absolute dose calibration for the universal wedge field. Minimal literature has been available until now. So in this paper, the dosimetric performance of universal wedge field measurement with MC3 was studied. Center axis (CAX) dose for different wedge angles, field sizes, different depths, and off‐axis doses along the wedge direction were measured. Non‐negligible errors were observed between ground truth (ion chamber measurements) and MC3 measurements. A dose rate correction strategy for the universal wedge field with MC3 was proposed, enabling its use in dosimetric verification of the universal wedge field.

## 2 MATERIAL AND METHODS

A 6 MV x‐ray beam provided by an Elekta Axesse LINAC (Elekta, Crawley, UK) was employed for all measurements. The LINAC was carefully commissioned using a water tank (BEAMScan, PTW, Freiburg, Germany) and appropriate ion chambers according to relevant standards.^19^ The percentage depth dose (PDD) at a depth of 10 cm with a 100 cm source‐to‐skin‐distance (SSD) amounted to 67.5%. According to the IAEA TRS 398 report,^20^ the absolute dose of the LINAC was calibrated such that 100 MU deposited 1 Gy at the maximum dose depth (1.5 cm). The accuracy of beam model in Monaco TPS (version 5.40.04, Elekta, Maryland Heights, USA) was carefully verified. The MC3 was employed for the measurements. The measurements under the same condition using the ion chamber (31021, PTW, Freiburg, Germany) calibrated at the National Institute of Metrology of China, were employed as the ground truth. Before measurement using MC3, ACF was determined following a stepwise calibration process according to the manufacturer's user manual. For absolute dose calibration, an ion chamber PTW 31021 and an electrometer (Tango, PTW, Freiburg, Germany) were first used to measure the absolute dose for a 10 × 10 cm^2^ open field under the standard conditions (listed in Table 1). The measured absolute dose was then used as the user‐input known dose to determine the DCF_open_ in the absolute dose calibration for MC3 under the identical condition. This DCF_open_ was used for the following measurements. All measurements were repeated three times.

**TABLE 1:** Standard condition of measurement.

Standard condition of measurement	MC3
SDD	100 cm
Intrinsic physical buildup	1.2 cm
Intrinsic water equivalent buildup	1.5 cm
Additional solid water buildup	8.5 cm
Total water equivalent buildup	10.0 cm
Intrinsic physical backscatter	1.8 cm
Intrinsic water equivalent backscatter	2.3 cm
Additional solid water backscatter	5.0 cm
Total water equivalent backscatter	7.3 cm
Spacing between adjacent detectors	7.07 mm
Dose	100 MU
Dose rate	^a^309 MU/min
Field size	10 × 10 cm

Abbreviations: MC3, MapCHECK 3; SDD, Source‐to‐detector‐distance.

^a^309 MU/min indicates that in our clinical routine, the intermediate dose rate was used for dose calibration. Considering the maximum dose rate of 6MV x‐ray for Elekta Axesse linac is about 600 MU/min and the stepwise dose rate variation pattern, we set 400 MU/min for dose rate before beam on, the actual dose rate was 309 MU/min during the measurement.

### 2.1 CAX dose for different wedge angle

Under standard measurement conditions, the CAX dose was measured for the wedge fields with different wedge angles (e.g., 15°, 30°, and 45°) using the PTW 31021 ion chamber. These angles are the same as those commonly studied in previous literature.^21^, ^22^, ^23^, ^24^, ^25^ Subsequently, the wedge fields were measured using MC3 with DCF_open_ under the same condition. The differences in CAX dose between the ion chamber and MC3 were compared for each wedge field.

### 2.2 CAX dose for different wedge field size

10 × 10 cm^2^ wedge field was taken as the reference field for dose calibration. To investigate the influence of field size, smaller (5 ×5 cm) and larger (15 ×15 cm) fields are selected. The CAX doses for wedge fields with different wedge angles (e.g., 15°, 30°, and 45°) and field size (5 × 5, 10 × 10, 15 × 15 cm^2^) were also measured and compared using ion chamber and MC3 under the standard condition. Again, the DCF_open_ was used as the absolute DCF for the measurements using MC3.

### 2.3 CAX dose at different depth

Measurements for the wedge fields with different wedge angles (e.g., 15°, 30°, and 45°) using ion chamber and MC3 were also carried out under the standard measurement condition but at different depths (5, 10, 15 cm). The CAX doses measured by MC3 calibrated with DCF_open_ were compared with those measured by the ion chamber.

### 2.4 Off‐axis dose along the wedge direction

Ion chamber PTW 31021 was used to measure the off‐axis doses along the wedge direction point by point for the 20 × 20 cm^2^ wedge fields with different wedge angles (15°, 30°, and 45°) under the standard measurement condition. The ion chamber was positioned perpendicular to the wedge direction to avoid stem effects. A 20 ×20 cm field was used for measurements to keep enough lateral scattering. Measurements began at the center of the field and moved to the next point by moving the couch every 1 cm interval along the wedge direction. The off‐axis doses along the wedge direction for the same wedge field were extracted from the MC3 measurement with DCF_open_. Only the off‐axis doses in the range of −9 cm to +9 cm along the wedge direction were compared to avoid the dramatic influence on the dose from the positioning error of the ionization chamber in the penumbra region.

### 2.5 Clinically relevant setups

Comprehensive wedge fields, which differ in wedge angle (15°, 30°, and 45°), field size (5 × 5 and 15 × 15 cm^2^), and measurement depth (5 and 15 cm) simultaneously from the absolute dose calibration condition, were measured using ion chamber PTW 31021 and MC3, respectively. The differences in the CAX doses between the measurements using the ion chamber and MC3 in the same measurement condition were compared. The average gamma pass rate differences between measurements using MC3 and the planned doses calculated by Monaco TPS using SNC Patient software were also compared under the same conditions.

### 2.6 Dose rate dependence correction

Average dose rate (ADR) dependence and instantaneous dose rate (IDR) are two kinds of dose rate dependence that can affect the sensitivity of the diode.^17^, ^26^, ^27^ Previous study^18^ showed that IDR correction was more effective for type 4 diode, so the IDR correction curve in the literature of Li et al.^18^ was adopted to correct the collected counts of each diodes in the MC3 measurement file. In the correction strategy proposed by Li et al., the total counts and irradiation times for each diode were first extracted from the .txt file. Then the ratio of the total counts and irradiation time, also known as dose per pulse (Dpp), was used to find the corresponding dose rate correction factor on the IDR correction curve. After being corrected by the dose rate correction factor, the corrected total counts were converted to the dose according to the manufacturer's manual.

However, this strategy cannot be straightforwardly applied to the correction of universal wedge field measurements as universal wedges achieve wedge field with an arbitrary wedge angle ranging from 0° to 60° by compositing open field and physical wedge field of 60° with a specific proportion. Consequently, the counts collected from the open field and the wedge field components should be corrected using their respective dose rate correction factors.

From the start of the measurement, SNC patient software (version 8.4.1, Sun Nuclear, Melbourne, FL, USA) recorded the cumulative counts of each diode and the beam status in the .mcm file every 50 ms. Based on the beam status, the cumulative counts of each diode can be categorized into five sequential phases: beam off, wedge beam on, beam off, open beam on, and beam off. During the first beam off phase, MC3 had already begun measuring but the LINAC had not yet been beam on. In the wedge beam on phase, the LINAC was beam on with the physical wedge inserted into the beam. The diodes recorded the counts from the wedge field component. In the second beam off phase, the LINAC was beam off while the physical wedge was moving out of the field. In the next phase, open beam on, diodes recorded the counts from the open field component. In the last beam off phase, the LINAC was beam off but the MC3 was still measuring. The counts collected by each diode during each beam off phase were mainly derived from background signals.

The raw counts collected in the wedge beam on and open beam on phases (RawCount_w,i_ and RawCount_o,i_) for diode i were separately calculated. For each component, Dpp values were determined and employed to calculate the dose rate correction factors from the IDR correction curve according to the method proposed by Li et al.^18^ After removing counts from the background signals, the $\mathrm{RawCoun}t_{w,i}$ and $\mathrm{RawCoun}t_{o,i}$ were corrected by individual dose rate correction factors, $y_{w,i}$ and $y_{o,i}$ (Equation 1 and 2). The dose rate correction factors were derived from IDR correction curve and Equation 3 in our previous work^18^

(1) $$\begin{array}{cc} & \mathrm{CorrectedRawCoun}t_{w,i}\\ & \;=\left(\mathrm{RawCoun}t_{w,i}-\mathrm{bc}g_{i}\times \;\mathrm{BeamO}n_{w}\right)/y_{w,i} \end{array}$$

(2) $$\begin{array}{cc} & \mathrm{CorrectedRawCoun}t_{o,i}\\ & \;=\left(\mathrm{RawCoun}t_{o,i}-\mathrm{bc}g_{i}\times \;\mathrm{BeamO}n_{o}\right)/y_{o,i} \end{array}$$

where $\mathrm{bc}g_{i}$ is the background calibration factor for the diode i; $\mathrm{BeamO}n_{w}$ and $\mathrm{BeamO}n_{o}$ is the total beam on time for physical wedge field of 60° and open field components.

Subsequently, the total corrected raw counts (CorrectedRawCount_i_), were the summation of CorrectedRawCount_w,i_ and CorrectedRawCount_o,i_ (Equation 3), and corrected using all other calibration factors (CF_all_) presented in the data file from MC3 (Equation 4). Finally, the counts after all correction (CorrectedCount_i_) for diode i are converted into the dose (DoseCount_i_) by multiplying the DCF (Equation 5).

(3) $$\begin{array}{cc} \mathrm{CorrectedRawCoun}t_{i} & =\;\mathrm{CorrectedRawCoun}t_{o,i}\\ & \;+\;\mathrm{CorrectedRawCoun}t_{w,i} \end{array}$$

(4) $$\mathrm{CorrectedCoun}t_{i}=\;\mathrm{CorrectedRawCoun}t_{i}\;\times \;CF_{\mathrm{all}}$$

(5) $$\mathrm{DoseCoun}t_{i}=\;\mathrm{DCF}\;\times \;\mathrm{CorrectedCoun}t_{i}$$

We developed an in‐house python program to deal with the dose rate correction and then reformatted into a .txt file which can be read by the SNC patient software automatically. The measured doses with dose rate correction were compared with the reference measured by ion chamber as well as the planned doses calculated by Monaco TPS using the SNC Patient software.

## 3 RESULTS

### 3.1 CAX dose for different wedge angle

**FIGURE 1:**
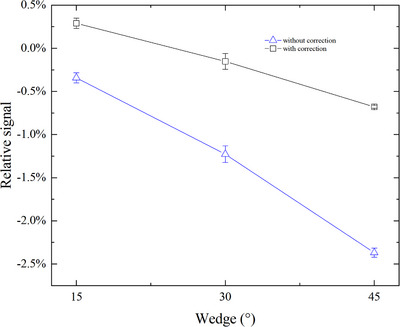
Relative signal in CAX dose for different wedge angle between MC3 and ion chamber without correction (blue curve) and with correction (black curve). CAX, central axis; MC3, MapCHECK 3.

Figure 1 shows the relative difference in CAX dose measured by the ion chamber and MC3 for wedge field with different wedge angle. Even under the same measurement conditions (field size, measurement depth, etc.) as absolute dose calibration, the CAX doses measured by MC3 with DCF_open_ are always smaller than those of the ion chamber. Moreover, as the wedge angle increases, a pronounced disparity, up to −2.4% for 45° wedge angle, emerges between the measurement derived from MC3 and ion chamber (Figure 1, blue curve). Wedge inserted into the beam would decrease the measured dose rate of CAX diode compared to the absolute dose calibration, and hence underestimate the CAX dose. So DCF derived from the open field is not appropriate for measuring the wedge field, especially for the wedge field with large wedge angle. After dose rate correction proposed by this study, the deviation reduces to the range from −0.7% to 0.3% for all wedge fields (Figure 1, black curve).

### 3.2 CAX dose for different wedge field size

**FIGURE 2:**
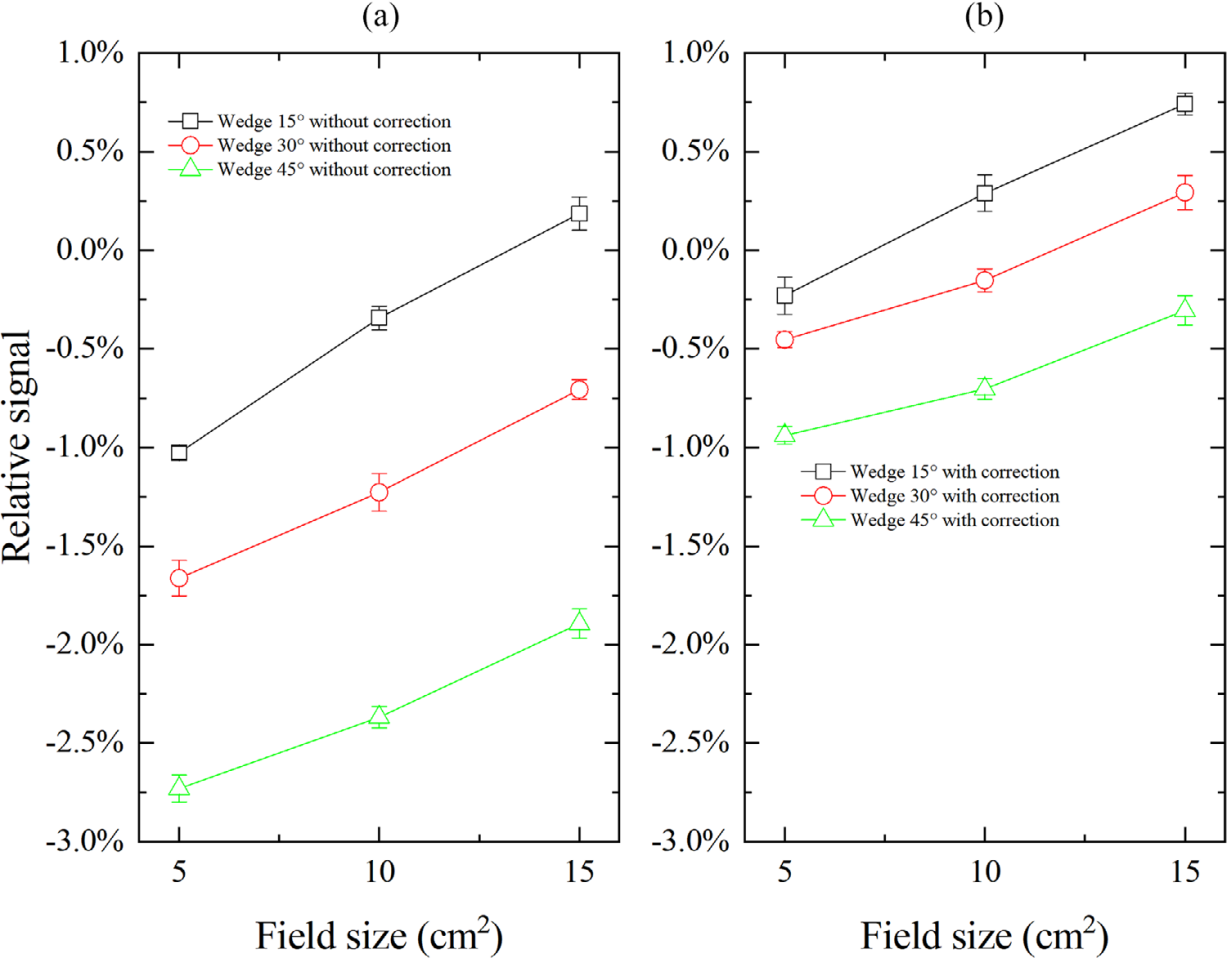
Relative signal in CAX dose for wedge field with different field size and wedge angle between MC3 and ion chamber without correction (a) and with correction (b). CAX, central axis; MC3, MapCHECK 3.

CAX dose deviation without and with correction between MC3 and ion chamber for wedge fields with different wedge angles and field sizes are shown in Figure 2a,b, respectively. For the wedge field with the same wedge angle, the deviations in the CAX dose for the wedge field larger than the reference field between MC3 and the ion chamber tend to decrease. On the contrary, a smaller wedge field would increase the deviation. For example, the deviations are −2.7%, −2.4%, and −1.9% for 45° wedge field with field sizes of 5 × 5, 10 × 10, and 15 × 15 cm^2^. After dose rate correction, the deviations for all wedge fields are within ± 1%.

### 3.3 CAX dose at different depth

**FIGURE 3:**
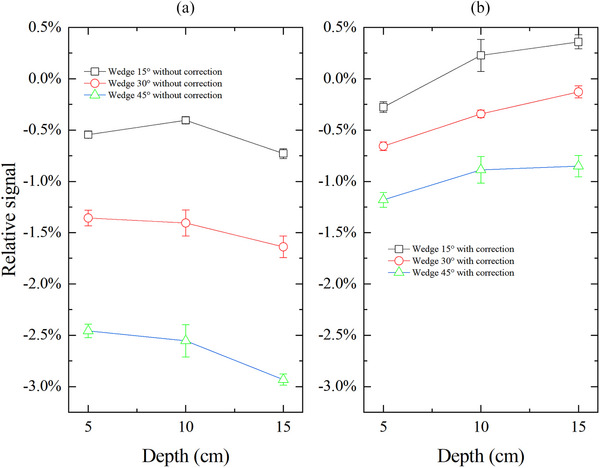
Relative signal in CAX dose for wedge field with different wedge angle at different depth between MC3 and ion chamber without correction (a) and with correction (b). MC3, MapCHECK 3.

As shown in Figure 3, the deviation in CAX dose between MC3 and ion chamber varies with the changes in measurement depth and wedge angle. In general, this deviation tends to increase as the measurement depth increases. Specifically, for the wedge field of 45°, the CAX dose measured at 15 cm depth using the MC3 with DCF_open_ introduces a deviation of approximately −2.9%. When measurements were performed for a 15° wedge field at a depth of 5 cm, a slightly bigger deviation was observed compared to the measurements taken at a depth of 10 cm. This may be due to the minute fluctuations (the variation coefficient for four consecutive measurements was less than 0.1%) in the output of LINAC during the measurement. However, after applying dose rate corrections, the discrepancies between the two measurement methods are consistently within ± 1.2%.

### 3.4 Off‐axis dose along the wedge direction

**FIGURE 4:**
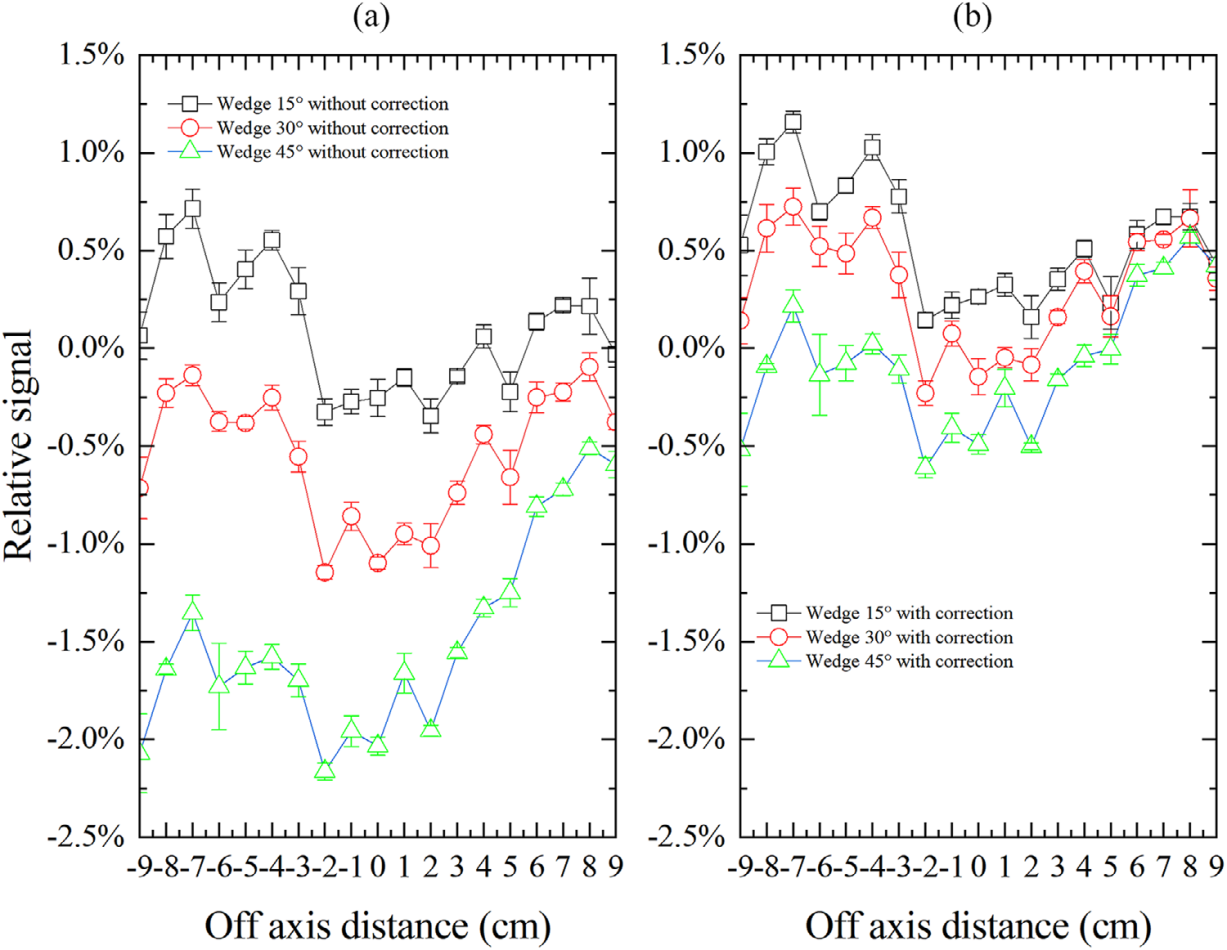
Differences in off‐axis doses along the wedge direction between MC3 and the ion chamber with and without correction. The positive direction of the off‐axis distance is the toe of the wedge, and vice versa.

As illustrated in Figure 4, we can also find that CAX doses measured by MC3 are smaller than those measured by ion chamber, and the deviation in CAX dose increases with the increase of the wedge angle. In addition, the deviation in off‐axis dose along the wedge direction between MC3 and ion chamber measurements shows some fluctuations, which may be due to the positioning error during the point‐by‐point measurement of off‐axis dose utilizing the ion chamber, as well as inherent fluctuations in the output of LINAC during the measurement. However, a noteworthy trend of the dose deviation along the wedge direction can be observed, with more pronounced deviations occurring on the heel side of the wedge compared to the toe side. For instance, for the 45° wedge field, the off‐axis dose deviation at −9 cm off‐axis distance (on the heel side) is about −2.1%, whereas the deviation at +9 cm off‐axis distance (on the toe side) is significantly small, at merely −0.6%. This will result in an overestimation of wedge angle based on the MC3 measurement.

After dose rate correction, the CAX dose deviation between MC3 and ion chamber is within ± 0.5%. Furthermore, the deviation in off‐axis dose along the wedge direction has been reduced to between −0.6% and 1.2%.

### 3.5 Clinically relevant setups

Table 2 represents the deviations in CAX dose for comprehensive wedge fields, whose wedge angle, field size, and measurement depth simultaneously differ from the absolute dose calibration condition. These represent more complex conditions encountered in clinical practice. The maximum deviation between the MC3 and ion chamber measurement is −3.2% without correction. However, after dose rate correction, it reduces to −1.2%. The deviations for the wedge field measured are all within the range of −1.4% to 1.2%.

**TABLE 2:** The deviation of the measured results from ion chamber with and without MC3 correction is shown when the measured depth, field size and wedge angle all deviate from the calibration condition.

Wedge angle (°)	Field size (cm^2^)	Depth (cm)	MC3 without correction	MC3 with correction
15	5 × 5	5	−1.0% ± 0.1%	−0.6% ± 0.1%
15	15 × 15	5	0.0% ± 0.1%	0.2% ± 0.1%
15	5 × 5	15	−1.3% ± 0.0%	0.0% ± 0.1%
15	15 × 15	15	0.2% ± 0.1%	1.2% ± 0.1%
30	5 × 5	5	−1.7% ± 0.2%	−0.9% ± 0.2%
30	15 × 15	5	−0.9% ± 0.1%	−0.2% ± 0.1%
30	5 × 5	15	−2.1% ± 0.1%	−0.5% ± 0.1%
30	15 × 15	15	−0.8% ± 0.2%	0.6% ± 0.1%
45	5 × 5	5	−2.8% ± 0.1%	−1.4% ± 0.1%
45	15 × 15	5	−2.0% ± 0.1%	−0.8% ± 0.1%
45	5 × 5	15	−3.2% ± 0.1%	−1.2% ± 0.1%
45	15 × 15	15	−1.9% ± 0.0%	0.1% ± 0.0%

Abbreviation: MC3, MapCHECK 3.

The gamma pass rates are summarized for different wedge fields in Table 3. Without correction, the gamma pass rate decreases with the wedge angle increase. For example, the average gamma pass rates are 98.8%, 95.8, and 89.9% for 15°, 30°, and 45° wedge field with different field sizes, respectively. The other point should be noticed that, when more parameters (wedge angle, field size, measurement depth, etc.) change compared to the calibration condition, the gamma pass rate will significantly decrease. For example, only the wedge angles differed from the absolute dose calibration for the wedge field in group A. The average gamma pass rates were 100%, 100%, and 99.7% for wedge field with wedge angles of 15°, 30°, and 45°, respectively. However, not only the wedge angle but also the field sizes and measurement depths differed from the absolute dose calibration for groups B and C, respectively. The average gamma pass rates decreased to 98.8%, 95.8%, 89.9%, and 100%, 98.2%, and 91.8% for 15°, 30°, and 45° wedge fields, respectively.

**TABLE 3:** Comparison of average gamma pass rate (criteria, 2 mm/2%) with and without dose rate correction.

	MC3 without correction			MC3 with correction		
	Wedge 15°	Wedge 30°	Wedge 45°	Wedge 15°	Wedge 30°	Wedge 45°
Group A	100.0% ± 0.0%	100.0% ± 0.2%	99.7% ± 1.0%	100.0% ± 0.0%	100.0% ± 0.0%	100.0% ± 0.0%
Group B	98.8% ± 0.3%	95.8% ± 0.9%	89.9% ± 1.0%	100.0% ± 0.0%	99.5% ± 0.3%	99.6% ± 0.0%
Group C	100.0% ± 0.1%	98.2% ± 0.3%	91.8% ± 0.5%	100.0% ± 0.0%	99.6% ± 0.1%	100% ± 0.1%

*Note*: Group A, B, and C represent three groups of wedge fields. Group A, 10 × 10 cm^2^ wedge field for each wedge angle measured at 10 cm depth; Group B, wedge fields for each wedge angle with different field sizes (5 × 5, 10 × 10, and 15 × 15 cm^2^) measured at 10 cm depth; Group C,10 × 10 cm^2^ wedge fields for each wedge angle measured at different depths (5, 10 and 15 cm).

Abbreviation: MC3, MapCHECK 3.

Upon dose rate correction, the reduction in CAX dose deviation is observed, accompanied by a substantial enhancement in gamma pass rates. Average pass rates for all wedge fields measured are greater than 99.5% after correction, even though a rigid criteria (2 mm/2%) is used. Especially for 45° wedge field with different field sizes and for 45° wedge field measured at different depths, the gamma pass rates improve from 89.9% to 99.6% and from 91.8% to 100%, respectively.

## 4 DISCUSSION

For the measurement of open field using MC3, the DCF is recommended derived from the measurement of reference open field and used for converting total collected counts into dose for each diode. However, this methodology is not appropriate for the measurement of the wedge field because the universal wedge field is composed of an open field and a physical wedge field of 60°in a certain proportion, and type 4 diodes equipped by MC3 has a more prominent dose rate dependence compared to previous diodes (type 1).^18^ The wedge inserted into the beam would significantly decrease the measured dose rate of diodes between calibration and measurement. So it would have a negative effect (underestimation) on the measured dose. If the counts collected by the diodes from the contribution of physical wedge field of 60° are converted into dose using the same DCF_open_, an error, up to −2.4% (see in Section 3.1), will be observed.

Other factors that may cause changes in the measured dose rate of diodes relative to the calibration conditions, such as the field size and measurement depth, can introduce errors to wedge field measurement. Larger field size or shallower measurement depth compared to the absolute dose calibration will increase the measured dose rate of diode, and hence have a positive effect (overestimation) on the measured dose. On the contrary, smaller field size or deeper measurement depth will decrease the measured dose rate, resulting in underestimation. The comprehensive effect of multiple factors (wedge angle, field size, and measurement depth) results in the deviation of the CAX dose as reported in Section 3.2, 3.3, and 3.5.

Another factor that may cause changes in the measured dose rate of diodes relative to the calibration conditions is the effective thickness of wedge inserted into the beam. The effective thickness of wedge inserted into the beam at the toe side is much less than that at the heel side. Therefore the measured dose rate of the diode at the toe side would decrease much less than at the heel side. This result in the finding in the Section 3.4, that more pronounced deviations occurring on the heel side of the wedge compared to the toe side.

In summary, the dose rate dependence of MC3 causes inaccuracies in the measurements of the CAX and axis dose for universal wedge fields. These inaccuracies will have a negative impact on clinical practice. In TPS commissioning, physicists may be puzzled when comparing this inaccurate measured dose distribution with the planned dose calculated by well commissioned TPS. More important, if physicists make a wrong decision to adjust the beam model in TPS based on these inaccurate data, the inaccuracy would ultimately introduced in the patients' treatment plan. In patient QA for wedge field plan, this inaccuracy in measured dose would decrease the gamma pass rate, especially for large wedge field, which is commonly used in whole‐breast irradiation.

One method to make the measurements of the wedge field using MC3 more accurate is to perform absolute dose calibration under the same conditions as the measurement (i.e., the same wedge angle, field size, and measurement depth) as much as possible. However, it is time‐consuming and not suitable for clinical application. Comparatively speaking, the correction strategy proposed in this study is more convenient. Once the IDR correction curve is established, the correction strategy proposed in this study has ability to correct all kinds of sources that would change the measured dose rate of diode. The methodology for absolute dose calibration used in open field measurement dose not require any alterations. The in‐house developed Python program will automatically correct the raw measurement results of MC3 and save them in a format recognized by SNC patient software for direct comparison with the planned dose just in few seconds.

After dose rate correction, not only does the CAX dose deviation reduce, but the gamma pass rates also significantly improve. The corrected measurement demonstrated a high level of agreement with the ground truth, ion chamber measurement. The deviation of CAX dose for all measured wedge field has been reduced to within ± 1.5%, and the average gamma pass rate has been enhanced to exceed 99.5% with a stringent criterion of 2 mm/2%.

There may be other factors which could potentially affect the accuracy of MC3 measurements, including directional dependence and energy response. These will be thoroughly investigated in the future to further improve the accuracy of MC3 measurement.

## 5 CONCLUSION

In summary, because of the more prominent dose rate dependence of type 4 diodes, MC3 is not suitable for universal wedge field measurement using the methodology for the open field measurement. The correction strategy proposed in this study is convenient and has ability to correct the dose rate dependence of type 4 diode during the measurement for universal wedge, making a good agreement with the ground truth.

## AUTHOR CONTRIBUTION

All authors have made substantial contributions to the work and development of this manuscript. All authors approved the manuscript. Linyi Shen, Mengyang Li designed and performed experiments, and completed the manuscript. Guiyuan Li, participated in measurements, scientific discussions, and manuscript writing. Xinyuan Chen, Shouping Xu: participated in scientific discussions and helped with manuscript writing. Jianrong Dai: supervised the study and helped with writing of the manuscript. Yuan Tian: performed measurement, analysis, and wrote the manuscript.

## CONFLICT OF INTEREST STATEMENT

The authors declare no conflict of interest.
